# Physical models can provide superior learning opportunities beyond the benefits of active engagements

**DOI:** 10.1002/bmb.21159

**Published:** 2018-10-03

**Authors:** Dina L. Newman, Megan Stefkovich, Catherine Clasen, Margaret A. Franzen, L. Kate Wright

**Affiliations:** ^1^ Thomas H. Gosnell School of Life Sciences, Rochester Institute of Technology Rochester New York 14623; ^2^ University of Wisconsin—Madison Madison Wisconsin 53706; ^3^ Drake University Des Moines Iowa 50311; ^4^ Milwaukee School of Engineering, Center for BioMolecular Modeling Milwaukee Wisconsin 53202

**Keywords:** Active learning, molecular biology, physical models, Central Dogma

## Abstract

The essence of molecular biology education lies in understanding of gene expression, with subtopics including the central dogma processes, such as transcription and translation. While these concepts are core to the discipline, they are also notoriously difficult for students to learn, probably because they cannot be directly observed. While nearly all active learning strategies have been shown to improve learning compared with passive lectures, little has been done to compare different types of active learning. We hypothesized that physical models of central dogma processes would be especially helpful for learning, because they provide a resource that students can see, touch, and manipulate while trying to build their knowledge. For students enrolled in an entirely active‐learning‐based Cell & Molecular Biology course, we examined whether model‐based activities were more effective than non‐model based activities. To test their understanding at the beginning and end of the semester, we employed the multiple‐select Central Dogma Concept Inventory (CDCI). Each student acted as their own control, as all students engaged in all lessons yet some questions related to model‐based activities and some related to clicker questions, group problem‐solving, and other non‐model‐based activities. While all students demonstrated learning gains on both types of question, they showed much higher learning gains on model‐based questions. Examining their selected answers in detail showed that while higher performing students were prompted to refine their already‐good mental models to be even better, lower performing students were able to construct new knowledge that was much more consistent with an expert's understanding. © 2018 The Authors. Biochemistry and Molecular Biology Education published by Wiley Periodicals, Inc. on behalf of International Union of Biochemistry and Molecular Biology., 46(5):435–444, 2018.

## Introduction

The “Central Dogma of Molecular Biology” refers to the concept of managing information flow in a cell, from storage in DNA through expression as a protein 1. The flow of genetic information is the cornerstone on which numerous topics and ideas in biology are built. This critical theme is included as one of the five Core Concepts of undergraduate biology education outlined by *Vision and Change* 2, as one of the four “Big Ideas” in the Advanced Placement Biology Curriculum Framework 3, and as the first learning objective in the Next Generation Science Standards for life sciences in high school (HS‐LS1‐1) 4. While biology experts can easily articulate the concepts and processes that encompass genetic information flow, biology learners struggle with these ideas 5, 6, 7, 8, 9, 10, 11, 12. When students think about the transfer of genetic information, many may remember superficial information about Gregor Mendel, Punnett squares, and pea plant phenotypes, but cannot grasp the underlying molecular processes and mechanisms that actually drive genetic information flow. Students may be able to recognize terms such as “transcription” and “translation” but often have poor mental models of these processes. When probed more deeply, they demonstrate little understanding of the structure of the biomolecular building blocks (e.g., nucleotides), the structure and function of the macromolecular products (e.g., RNA), or the molecular interactions that facilitate these processes (e.g., complementary base pairing of incoming nucleotides to the DNA template) 9, 13. This lack of understanding ultimately creates a shaky foundation for molecular biology knowledge, making it more difficult to productively scaffold higher‐level concepts related to genetics and gene expression in the future.

DNA, RNA and proteins are complex biomolecules that are both incredibly small (generally not visible with a microscope), yet extraordinarily large (often thousands or millions of subunits). Research has demonstrated that students have particular difficulty bringing in and using the molecular/submicroscopic scale when wrestling with complex ideas about genetic information 14, 15, 16. Learners, then, must rely on visual representations in textbooks or online resources to help them “see” molecules and molecular level interactions that drive processes such as replication, transcription and translation. While it is not problematic for experts to productively interact with discipline‐specific drawings or illustrations, students often lack representational competence, making many visual resources less‐than‐ideal for learning 17, 18, 19, 20, 21, 22, 23. Strategies that explicitly make the “invisible” more visible to learners may help students overcome some of the challenges associated with learning concepts of genetic information flow.

Just as certain topics may be more or less challenging for undergraduate students to learn, certain pedagogies are more or less effective in promoting learning in the undergraduate science classroom. Although lecture‐only pedagogies have been shown to be relatively ineffective for student learning, many college STEM instructors still use a lecture‐only approach in their teaching 24, 25, 26, 27. Numerous studies in the STEM education research literature have demonstrated that active‐engagement learning strategies result in higher learning gains and reduce the chance of failure compared with lecture‐only approaches 25, 28, 29. Active‐engagement strategies encourage students to create their own knowledge in the classroom setting and often include peer discussion. Some common examples of active‐learning lessons are: think‐pair‐share discussions, clicker question debates, case study analyses, group problem sets, designing experiments, devising models to explain phenomena, and using physical models to explore concepts.

Constructing and using models is a practice used by scientists to ask and answer questions about the natural world. Models facilitate discussion and discovery by providing scientists a shared resource as a baseline or starting point. It is not surprising, then, that recent educational reform initiatives in K‐12 30 and higher education 2 call for the increased use of models and model‐based activities in STEM classrooms. There is a growing body of literature supporting the use of models to improve STEM learning, but more research focusing on how students use models and representations to learn is needed 27. For example, when used in a sophomore‐level honors introductory biology course, physical models of biomolecules deepened the knowledge of structure–function concepts as evidenced by higher quiz scores and self‐reported learning gains 31. Interestingly, it was the female students who experienced higher learning gains compared with control female students who did not use the models. While additional research is needed, the authors suggested the effect could be due in part to the finding that females on average have lower spatial perception and mental rotation skills than males 32 which the models helped ameliorate. Likewise in an organic chemistry class, activities involving ball‐and‐stick physical models in conjunction with computer‐generated 3D models yielded higher student scores on a post‐test than two‐dimensional (2D) textbook representations, or either the ball‐and‐stick or computer models alone 33. Work presented by Wu *et al*. also underscored the importance of allowing learners to explore multiple representations and build and manipulate molecular models using a computer‐based visualization tool called eChem 34. In this study, high school chemistry learners demonstrated improved understanding of chemical representations and were highly engaged with the modeling visualization tool. The authors also stated the “*findings suggest that models can serve as a vehicle for students to generate mental images…”* lending support to the idea that models may provide students with a tool on which to scaffold and build new knowledge.

Because we have been using physical model‐based activities and the CDCI assessment in a Cell and Molecular biology course, we decided to undertake a retrospective study using three years’ worth of assessment data. We asked if these model‐based activities were a superior instructional tool compared with other active‐engagement strategies when teaching concepts related to the Central Dogma of Molecular Biology. In this article, we present compelling evidence that physical model‐based activities focusing on biomolecules and information flow produce higher learning gains on Central Dogma related concepts than do other active‐engagement strategies (non‐model‐based activities). Furthermore, we describe how features of model‐based activities may reduce cognitive load to improve learning and discuss how model‐based activities may align with frameworks of cognitive sciences to create optimal learning environments for students.

## Methods

### Model‐Based Activities

Several physical models of biomolecules and processes were used as the foci of activities in a Cell and Molecular Biology course to teach Central Dogma concepts. Except where indicated, each of these models was purchased from 3D Molecular Designs (http://www.3dmoleculardesigns.com/3DMD.htm), but they are also available from the Milwaukee School of Engineering Model Lending Library (cbm.msoe.edu/lendingLibrary/). Descriptions of the relevant models are found in the Appendix.

Activities for groups of three to five students were designed to go with each model, including instructions for manipulating the models and questions to answer, in order to scaffold student learning. The course, which is designed for second‐year undergraduates in a biology‐related major, was taught in nine sections of 40–55 students, with three different instructors over a 3‐year period, using the same materials each time. During these student‐centered lessons, the instructor and an undergraduate learning assistant circulated around the room, asking probing questions of each group to promote deeper understanding of the concepts.

### Non‐Model‐Based Activities

Although models were used extensively in the Cell and Molecular Biology course, not every topic had a model to go with it. Nevertheless, all class sessions were based on active learning strategies. Descriptions of non‐model‐based activities can also be found in the Appendix.

### Class Description

All data were collected with Institutional Review Board approval. The assessment data were collected in a 200‐level Cell and Molecular Biology course, which is a requirement for a number of biology‐related majors. A total of 411 students completed the course during the Fall semesters of 2015, 2016, and 2017. Students were divided into three class sections of approximately 40–50 students per semester, and each section was supported by a Learning Assistant 35. Students worked in groups (3–6 students per group, depending on the activity) during model and non‐model based activities; 75% of the time students self‐selected into groups, 25% of the time the instructor randomly sorted students into new groups (i.e., students were given index cards with a group designation on it). In addition to the instructor, a Learning Assistant circulated around the classroom to help facilitate group discussion and help guide students if questions arose. Three instructors were involved in the course during the time described. Pre‐/post‐matched assessments were available for 300 students.

### Pre/Post CDCI Testing

Students enrolled in the Cell & Molecular Biology course were given the Central Dogma Concept Inventory (CDCI) at the beginning and end of each semester 9. The CDCI is a validated, 23‐question, multiple select instrument that focuses on concepts related to the Central Dogma of Molecular Biology. Students in the course had no prior knowledge that the CDCI would be deployed as a pre or post‐course assessment, and thus completed no special preparation or review before the post‐test was given.

Before any analysis of data, researchers (LKW and DLN) did a preliminary analysis, parsing the CDCI questions into two groups: 1) questions in which the underlying concepts aligned with a model‐based activity used in the class and 2) questions in which the concept did not align with a model‐based activity. The CDCI questions and the list of models used in the course were presented to a group of eight biology faculty who were experienced with most of the model‐based activities but were not directly involved in the study. Feedback from the eight scientists agreed with the authors’ alignment. Approximately half of the questions (12) related to a model‐based activity and half (10) related to non‐model‐based activities; one CDCI question was excluded from the analysis due to ambiguity of its classification.

Students who did not complete either the pre or post‐test (due to late enrollment, absences or course withdrawal) were excluded from analysis. Data were analyzed in the following ways: 1) Learning gains of whole question score: questions were only marked as “correct” when they chose the correct combination of correct answers (no partial credit). Normalized learning gains were calculated from the pre and post percentages as (post − pre)/(1 − pre) 28. 2) Differences in correct/incorrect responses: partial scores were calculated by taking all responses into account, as each multiple select question had 1–5 “correct” options and 0–4 “incorrect” options (42 total correct and 51 total incorrect). The percentage of correct and incorrect choices by each student was calculated, and pre and post test scores were compared. 3) Quartile analysis based on whole question pretest score: the 300 students were ranked by pretest score to form four quartiles of 75 students each. When it was necessary to split a group of students who had the same whole question score, they were ranked by their partial credit score.

Paired *t*‐tests were used to determine the significance of differences observed between questions that related to a model and those that did not.

## Results

Students enrolled in the Cell & Molecular Biology course were exposed to many different types of active learning strategies as they learned about Central Dogma concepts. Since none of the class meetings was devoted entirely to lecture, we were able to test whether model‐based activities were superior to other active‐learning pedagogies for teaching Central Dogma‐related concepts. Table 1 illustrates the CDCI questions that aligned with one or more of the model‐based activities in class and those that aligned with other active‐engagement strategies.

**Table 1:** Alignment of CDCI questions with classroom activities

CDCI V5	Major concept	Model that addressed concept
Q1, Q10, Q15	Mechanism of RNA synthesis	Flow of Genetic Information Kit^©^ ‐ Transcription
Q2, Q14, Q16, Q20	Mechanism of protein synthesis	Flow of Genetic Information Kit^©^ ‐ Translation
Q5, Q6, Q7, Q21	There are multiple types of information encoded in DNA that may be used at different times	Bioinformatics Map of the β‐Globin Gene^©^, Splicing model
Q17	Macromolecules are comprised of specific building blocks (differentiate between these categories)	Amino Acid Starter Kit^©^, DNA Discovery Kit^©^, Flow of Genetic Information Kit^©^

CDCI V5	Major concept	Non‐model based activity that addressed concept
Q3	Macromolecules are comprised of specific building blocks (compare chemical structures)	Clicker question and annotation of chemical structures.
Q4	Mechanism of protein synthesis (RNA as a catalyst)	Online splicing animation and follow‐up discussion questions
Q8, Q9	DNA is permanent information storage and products (RNA and proteins) are synthesized when needed (differential gene expression)	Lac operon (online) simulation followed by clicker and conceptual prediction questions. Drawing activity, linking ligand binding to gene expression.
Q11, Q12	DNA is permanent information storage and products (RNA and proteins) are synthesized when needed (functions of DNA and mRNA)	pGLO^®^ laboratory‐based project (multiple weeks) and annotations of diagrams of the arabinose‐inducible expression system.
Q13	Mechanism of RNA synthesis (different kinds of RNA)	Clicker and conceptual problems, annotation of chemical structures.
Q19, Q22, Q23	Although mistakes can occur in any CD process, mutations are permanent changes in the DNA	Clicker questions and prediction questions. Theoretical gene problems (descriptions or schematic gene map diagrams) plus conceptual/prediction questions.

**Figure 1:**
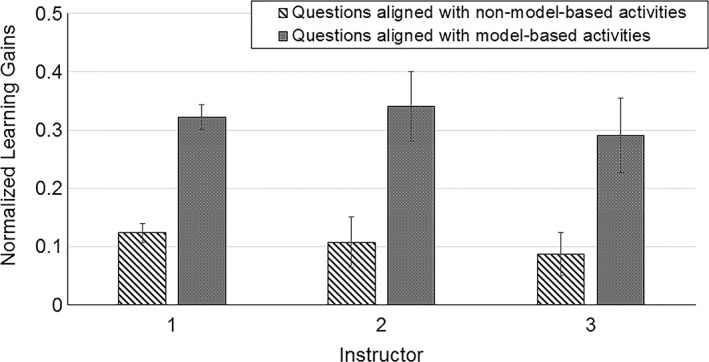
Students made significantly higher normalized learning gains on questions aligned with model‐based activities (dotted bars) than on questions aligned with other types of activities (striped bars) for all three instructors who taught the course over three years (instructor 1: *n = * 222 students from 6 sections; instructor 2: *n = * 29 students from 1 section; instructor 3: *n = * 49 students from 2 sections). Questions were scored as all right or all wrong for this analysis (no partial credit). Paired *t*‐test for each instructor, *p* < 0.001. Error bars are SEM.

Whole question scores (no partial credit) were used to calculate normalized learning gains for each CDCI question. In order to test our hypothesis that model‐based activities helped students learn more than non‐model based activities, we grouped CDCI questions into those that aligned with model‐based activities and those aligned with non‐model based activities. As illustrated by Fig. 1, regardless of the individual instructor, students made significantly higher gains on concepts that were taught using model‐based activities compared with concepts taught using other active‐engagement strategies. To investigate possible gender differences (i.e., did the use of physical model‐based activities help male students more than female students, or female students more than male students?) we compared learning gains for male (*n = * 103) and female (*n = * 196) students for concepts that were taught with and without models. Although males had higher scores overall, both groups benefited equally from using models (very large effect size, Cohen's *d* = 1.1 for males and 0.99 for females).

**Figure 2:**
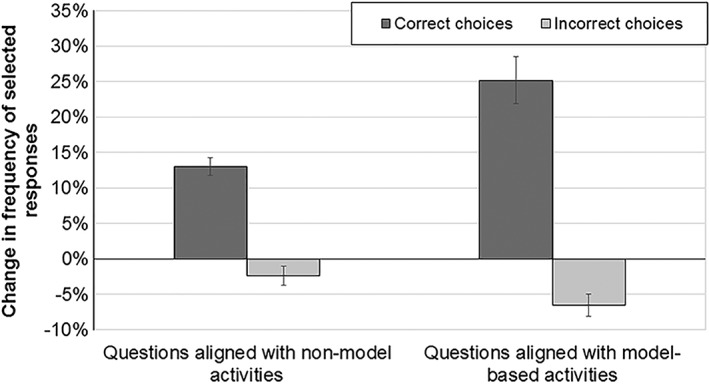
Students improved by choosing significantly more correct responses and significantly fewer incorrect responses on all questions, but they made significantly bigger changes on the multiple select questions aligned with model‐based activities compared with those questions aligned with other types of activities. Error bars are SEM.

We then leveraged the format of the CDCI to do a more thorough analysis on student responses, as we also were interested to learn if students made partial gains on assessment questions. In other words, did students choose more correct choices and fewer incorrect choices pre to post? For this analysis we calculated the change in frequency of correct and incorrect responses for CDCI questions aligned with model‐based activities compared with CDCI questions aligned with non‐model based activities. Figure 2 illustrates a significant difference in how students answered the two groups of questions. While, overall, students did choose more correct responses and fewer incorrect responses on the post‐test, the changes were more dramatic in CDCI questions aligned with model‐based activities; students chose even more correct responses and fewer incorrect responses. To investigate any gender‐specific results, we also compared the change in frequency of selected responses in male students to the change in frequency of selected responses in our female students. There were no significant differences in the percent of correct or incorrect answers pre to post for males compared with females. Thus, similar to the whole‐question analysis, we concluded that model‐based activities benefitted both male and female students equally.

**Figure 3:**
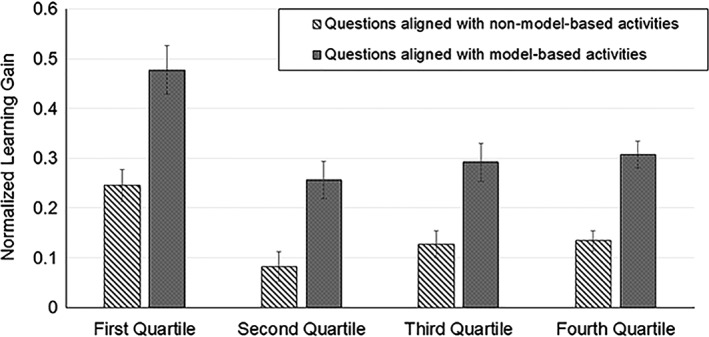
Students in all quartiles showed higher learning gains on questions aligned with model‐based activities (dotted bars) than on questions aligned with other types of activities (striped bars). Quartiles were determined by scores on the entire pretest (first quartile = highest scoring students; 75 students per quartile). Questions were scored as all right or all wrong for this analysis (no partial credit). Paired *t*‐test for each quartile, *p* < 0.001. The first quartile shows significantly higher normalized learning gains for all types of questions, but the other quartiles show no significant difference from each other in normalized learning gains on model‐based questions. Error bars are SEM.

We were also interested to know if the model‐based activities had positive impacts for all students, or only students in certain ability quartiles. We retroactively sorted students into ability quartiles based on the pre‐course score on the CDCI tool and calculated normalized learning gains for each quartile. Students in the first quartile outperformed students in the other quarters dramatically, on both pre‐ and post‐tests (Fig. 3). However, all quartiles made significantly higher gains on questions aligned with model‐based activities compared with those aligned with non‐model‐based activities. One explanation for this phenomenon is that only students in the first quartile are likely to get many whole questions right, while lower performing students are more likely to get part of the question correct without getting the whole question correct. Thus, it appears that there is a big difference between the first quartile and other students. However, smaller learning gains based on whole questions does not necessarily mean that the lower‐performing students are not learning as much—they may be moving from more incorrect to more correct.

**Figure 4:**
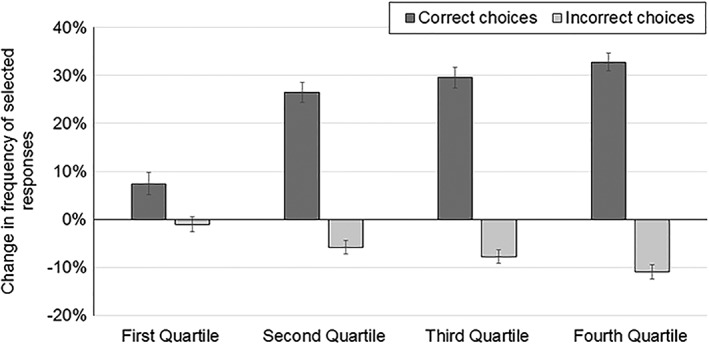
Students improve on questions aligned with model‐based activities by choosing significantly more correct responses in all quartiles and significantly fewer incorrect responses in the lower three quartiles. Quartiles were determined by scores on the entire pretest (first quartile = highest scoring students; 75 students per quartile). Error bars are SEM.

To explore the hypothesis that lower quartile students made learning gains without getting whole questions correct, we calculated the average changes in frequency of selected responses for model‐aligned CDCI questions for all quartiles of students (Fig. 4). Here our analyses revealed the opposite trend of the whole question analysis: students in the first quartile changed very little compared with students in the other three quartiles. Students in the lower quartiles made the greatest changes in how they answered each assessment question. For example, the change in frequency (pre to post) in the overall percentage of correct responses chosen was less than 10% for first quartile students but 32% for the fourth quartile.

## Discussion

Compared with lecture‐only pedagogies, active‐engagement strategies yield higher learning gains and can help reduce achievement gaps in STEM courses 25, 36, 37. Active‐engagement strategies are almost universally better than a lecture‐only approach, because they generally engage students in thinking, not just doing (“hands‐on, minds‐on learning”) 38. Most published work, however, has focused on comparing active‐learning courses to traditionally taught courses and not on comparing different active‐engagement strategies within the same course.

In this study, we have taken a slightly different approach. Using the validated published Central Dogma Concept Inventory tool 9, we showed that students make significantly higher learning gains on assessment questions that align with a physical model‐based activity compared with assessment questions that align with a different (non‐model based) active‐engagement strategy like clickers or peer discussion problems. While no one test can assess all student learning objectives, we find the results from the CDCI tool to be especially encouraging because concepts related to the Central Dogma and information flow are difficult for many students 9, 11, 12, 13, and this works suggest a strategy that might be very useful to other instructors. Many different learning theories suggest that models and model‐based activities should be effective learning tools. While more research is needed to determine how and whether they apply to the context of molecular biology education, we present an overview of the most pertinent theories to this research.

### Constructivism

The basic tenet of constructivism is that students learn best when they construct their own explanations through guided activities 39. The model‐based activities used in this study are dynamic, physical tools that allow refinement and reorganization of students’ mental models of molecular processes. Most college biology learners enter a molecular biology course with some knowledge of protein translation, for example, but have a faulty mental model of how the process actually works, especially on the molecular level. Students may know that amino acids become covalently linked together during protein translation and that the sequence of codons somehow directs the process, but are not sure how the mRNA really directs this process. Or students may understand tRNAs as “transfer” or “adaptor” molecules, but think tRNAs interact outside of the ribosome. During a model‐based activity, students can explore the structures and interactions that drive translation without being “told” how the process works. With guidance, students build their own model of translation and, thus, construct their own knowledge about the process.

### Zone of Proximal Development

In addition to models helping students correct their faulty mental models, psychologists know there is an intrinsic social quality in learning and cognitive development. Vygotsky's theory of cognitive development suggests learning occurs in the *zone of proximal development; a* measurement between a student's ability to solve a problem independently compared with instructor/peer guidance 40. In other words, students require scaffolding to learn new things and cannot incorporate ideas that are too far removed from prior knowledge. Since experiences, ideas, and foundational knowledge vary among incoming students, it can be challenging for instructors to create lessons that build upon existing knowledge in a productive way.

The shaky foundational knowledge and incorrect mental models of Central Dogma concepts held by many students prevent them from productively incorporating new material. If a student cannot visualize the process of transcription, for example, how can they conceptualize and understand the more complex idea of gene regulation? Well‐designed models in biology help give students a “starting point” on which to scaffold new information and ideas which also help correct or fill in gaps of faulty incoming mental models. By constructing a model of RNA transcription with peers, for example, students are able to “see” and verbalize the steps of the process, especially at the usually‐hidden molecular scale, and build upon the new (and correct) model of the process as they attempt to learn new things. For instance, the Flow of Genetic Information Kit© (FGIK) transcription activity includes an RNA polymerase structure that facilitates proper construction of the mRNA and prevents RNA from being built backward (in the incorrect 3′–5′ direction). Similarly, in the FGIK Translation activity, as students push the mRNA through the ribosome, foam bumpers cause the incoming amino acid to physically touch the growing polypeptide chain allowing students to properly connect them, representing peptide bond formation. This idea has been articulated by Yelland and Masters 41, who proposed manipulative models could serve as cognitive scaffolds.

### Reduction of Cognitive Load

Cognitive load theory dictates that the cognitive architecture of humans limits the conditions that will be ideal for learning 42, 43. Most people have limited working memory and can only hold 5–9 pieces of information at the same time 44. In order to learn something deeply, information must be transferred from short‐term working memory and integrated into a schema, a larger knowledge structure in the long term memory 45, 46. If instructors are to develop materials and activities to promote deeper learning, they must be cognizant not to overload students’ working memory with too many pieces of information. The manipulative models used in this study may help reduce cognitive load by providing learners with a physical 3D structure to look at, point to and manipulate. The presence of a physical model removes the need to “hold” that piece of information in the minds as a mental model. Once students modeled the process of protein translation, for example, they no longer have to rely on their memory for the structure and role of tRNAs, the difference between codons/anti‐codons and the direction of polypeptide chain synthesis because these features are part of the dynamic model. We suggest that having the physical model to refer to may free up space in their working short‐term memory.

### Shared Mental Model

As students work with the model, they refine and match their mental models to fit the physical model more closely and thus can refer to shared ideas during discussion and problem solving. The adage “a picture is worth a thousand words” reflects of the value of a common mental model between speaker and listener. Physical, interactive models become the embodiment of a shared mental model while also allowing for a dynamic discussion of interactions and processes.

### Implications for Teaching

Learning, like scientific practice, assimilates observations into a cohesive schema; as additional information is gathered, the schema is modified to result in iterative knowledge refinement. While non‐model active engagement strategies can also align with theories of cognitive science that promote learning, model‐based activities may be superior for helping students learn topics related to molecular biology and genetic information flow. While performance on assessment questions is only a proxy for measuring learning, our data consistently show that students make higher gains on concepts that were taught using a model compared with concepts taught with other active engagement strategies. In contrast to a recent study by Forbes‐Lorman 31, which showed that the use of physical models benefitted female students more than male students, in our study male and female students benefitted equally. However, the two studies were quite different in scope and approach. The interventions tested in the Forbes‐Lorman study were designed to teach honors‐level biology students about one particular protein and encompassed one class period. Our study retrospectively examined data from a whole semester and involved multiple model‐based activities that focused on molecular processes and interactions. Thus, while some models or model‐based activities, in certain situations, may be more beneficial to female students compared with male students, we cannot support a gender‐specific benefit with our dataset.

While the highest performing students in the class show the greatest normalized gain on the multiple select assessment questions, the lowest performing students make the greatest absolute gains. Viewed through the lens of the expert‐novice continuum 47 we hypothesize the following. The model‐based activities helped high performing students *refine* their mental models of Central Dogma processes and concepts and made them even more expert‐like. Students in the lowest two quartiles, who entered the class with mostly incorrect ideas (or no knowledge) about Central Dogma processes, constructed correct mental models about these processes. Students in this group may have not had the time to refine their own models all the way to the expert side of the continuum, but still made significant gains in their learning. The model‐based activities described here seemed to benefit all students, regardless of what knowledge they entered the course with. One caveat of this study is that it was retrospective rather than prospective. Thus, we did not create parallel activities to target the same concepts with and without models. In contrast to the traditional case‐control study, which compares the performance of different students on the same concepts, we compared the performance of the same students on different concepts (i.e., each student is a control for themselves). We used normalized learning gains to negate the effect of differences in question difficulty, so we are still able to conclude that students learned more when models were used. There are many resources that help good students get better or provide remedial help for struggling students, but well‐designed physical models are particularly beneficial because they seem to support learning of all students, regardless of status.

## Activity Descriptions

### Models Used in the Class

Except where indicated, physical models were purchased from 3D Molecular Designs; they are also available from the Center for Biomolecular Modeling's Lending Library (http://cbm.msoe.edu/lendingLibrary/index.php). Groups of three to five students worked through in‐house‐designed activities that guide them through important features and lead them to essential concepts.

1. **Flow of Genetic Information Kit^©^ (FGIK)**: https://www.shop3dmoleculardesigns.com/Flow-of-Genetic-Information-Kit-p/fgik.htm
  This kit is intended to illustrate the processes of DNA replication, transcription, and translation. In this study, only the transcription and translation activities were used. Each of these processes is modeled in a dynamic fashion, using foam pieces to represent the biomolecules. Enzymes are constructed from foam attached to plasitcized paper, which provide channels for chains of foam nucleotides to pass through.
  - Foam nucleotides used in all three parts are designed so they can be linked together with an arrow‐shaped peg that points in the direction of chain synthesis (5′ → 3′). Base pairs between chains are facilitated with weaker interlocking connections. Deoxyribonucleotides are differentiated from ribonucleotides by the shapes of their sugars.
  - For the process of transcription, double‐stranded DNA is threaded into an RNA polymerase, which has a wedged channel to separate the DNA strands. One strand is used as a template to build RNA. A second wedge separates the DNA from the growing RNA strand, which exits via a bridge channel, and the two DNA strands rejoin and exit via the first channel.
  - For the process of translation, single stranded RNA is threaded through the ribosome's channel, where they interact with charged tRNAs. The foam tRNA models have a three base anticodon that interlocks with the matching codon on one end, and an amino acid binding site at the other end. Amino acid pieces interlock to build the growing peptide chain and then are separated from the tRNAs by a wedge as they exit the ribosome. A termination factor binds to the stop codon to allow for protein release.
2. **DNA Discovery Kit** ^**©**^: https://www.3dmoleculardesigns.com/Teacher-Resources/DNA-Discovery-Kit.htm
  This kit is intended to illustrate the structure of the DNA double helix. It contains CPK‐colored plastic pieces of nucleotides (deoxyribose, nitrogenous bases, phosphates) that fit together with pegs and holes, designed so that incorrect connections are not possible and the geometry of the chemistry is revealed. Watson‐Crick base pairing is facilitated with magnets, also designed so that incorrect connections are not possible. When multiple nucleotides are connected together, the double helix forms, with easily discernable major and minor grooves. The kit also contains the Plectonemic DNA Model, which includes a pair of mini‐toobers and a plastic form to wind them around to make a double helix. This model of the double helix is useful for demonstrating the need for helicase in replication (i.e., the strands cannot separate without unwinding).
3. **Bioinformatics Map of the β‐globin Gene** ^**©**^: https://www.shop3dmoleculardesigns.com/Map-of-the-Human-Globin-Gene-p/bggm.htm
  This model consists of the entire genomic sequence of the human β‐globin gene printed on laminated paper, approximately 15 feet in length. Both strands of DNA and its translation in all three reading frames are shown. Examination of the sequence will identify features of the gene (introns, exons, TATA box, transcriptional and translational start and stop sites, all three reading frames, etc.). An instructor's version has all of these features, along with common mutations, marked on it.
4. **Splicing Model** (homemade, not available through the Lending Library): This paper model is used to illustrate the looping out of introns during the splicing process. It consists of a strip of mRNA sequence, and students use their fingers and thumbs to mimic how snRNPs (protein/RNA complexes that held mediate the splicing process) help bring distant sites on the mRNA close together, scissors and tape to model breaking and formation of new bonds in the sequence at the appropriate points. Students are also asked how various mutations may impact the splicing process. *(Readers can contact authors if interested in using this model in their own classrooms.)*

### Non‐Model Based Activities

1. **Conceptual/prediction questions**: Students would be given a worksheet with a description of a phenomena (and possibly a diagram or illustration) and a series of questions to answer working with their peers. For example, students compare and contrast five different DNA structures that each have an unreplicated portion of the double‐stranded structure (e.g., 3′ overhang, a single‐stranded circular DNA molecule). Students are asked if each structure would require a primer and/or the action of DNA ligase to complete replication and asked draw the replication process as they understand it would occur. In another example students are presented with information about an unfamiliar bacterial operon and predicted which regions corresponded to the promoter, structural genes, and regulator gene using mutation data.
2. **Clicker questions**: Students would be presented with a clicker question (with multiple choice responses) presented on a PowerPoint® slide. Students were encouraged to discuss ideas with their nearest peers and vote on the best response. In instances when two answers emerged as the two “winners” (and only one of the responses was correct) students were encouraged to turn to students sitting in front or behind them (so they were interacting with new students) and explain their reasoning for choosing the response they did and come to consensus with their new group. For example, students were presented with various visual representations of double‐stranded DNA helix structures with different shapes representing different things in each model (e.g., spheres represented atoms on one structure, but represented chemical groups in another model. Different color schemes were used depending on the representation). Students were asked to decide what shapes and or color represented in some of the models using clickers. In another example, students were presented with list of elements found in a typical eukaryotic gene (promoter, exons, and introns). Students were asked to think about the processes of DNA replication and transcription and then decide which elements were used as template in both process and which were used as templates in one process (e.g., DNA replication but no transcription). In a third example students were asked to predict which of the following cell types (from a list) from the same person would include a gene for a liver‐specific enzyme.
3. ***Lac* operon online simulation and activity**: Students were asked to complete the Phet online *lac* operon simulation called “The Gene Machine” (https://phet.colorado.edu/en/simulation/legacy/gene-machine-lac-operon) before class. Students were also asked to answer questions about each of components of the simulation such as, “*This [image inserted] is the protein product of the lacZ gene. What function does it carry out*?” Students were also asked to determine if the regulatory components (the *lac* promoter and the *lac* operator) were comprised of DNA, RNA, or protein. During class students confirmed their answers through class discussion or simple clicker questions. After more in‐class discussion students were asked to draw the interactions occurring at the lac operon during different conditions (e.g., high glucose and no lactose high glucose and high lactose, no glucose and high lactose) and answer additional prediction‐type clicker questions such as “*Imagine a laboratory strain of Escherichia coli that contained a mutation in the lac operator site. This mutation resulted in a change in the DNA sequence so that LacI could no longer bind. What happens when there is high glucose and no lactose in the growth media?”*
4. **pGLO^®^ laboratory project**: Students in the Cell and Molecular biology course completed a laboratory‐based project that allowed them to investigate macromolecules of the Central Dogma (DNA, RNA, and protein) in *E. coli* cells transformed with the pGLO^©^ plasmid (Biorad). The pGLO^©^ plasmid contains part of the inducible Arabinose operon which acts as a control switch for GFP expression. During the multiweek project students manipulated growing conditions of transformed *E. coli* cells and then detected the gene (DNA) for Green Fluorescent Protein (GFP), GFP mRNA, and GFP protein in control and experimental conditions. In addition, students completed an online quiz to correctly decipher the symbols used in visual representations of operons and completed several laboratory writing assignments describing results of experiments.
5. **Theoretical gene problems**: Students were asked to draw a schematic diagram of a eukaryotic gene based on descriptive text (e.g., Size of processed mRNA was given, positions of start and stop codons were given, etc.). Students were then asked to figure out what percentage of the mature mRNA was used as template during the process of translation and predict the size of the translated polypeptide in the case of a particular mutation. In a different activity, students were asked to predict sizes of protein products (by drawing bands on a western blot) based on various mutations. Students were also presented with a scenario in which a gene contained a nonsense mutation in an exon; students were asked to predict the size of the mRNA and protein products of the wild‐type versus mutant allele.
6. **Online splicing animation**: Students watched various online splicing animations (e.g., one from the DNA Learning Center https://www.dnalc.org/view/16938-3D-Animation-of-RNA-Splicing.html), and answered questions (that were part of a pre‐class assignment but also discussed during class) about the role of snRNAs and snRNPs.
